# Antimicrobial Resistance Patterns and Clinical Outcomes of Gram-Negative Bloodstream Infections in Saudi Arabian Hospitals: A Systematic Review and Meta-Analysis

**DOI:** 10.7759/cureus.102077

**Published:** 2026-01-22

**Authors:** Ayman Alqurain, Manal Hakami, Ahmed Osman Hassan Ali, Reham Abd El Rahman, Arwa F Al-Nefaie, Abdulrahman A Alahmari, Bandar S Alshreef, Mira A Al-Ghamdi, Khader M Alqarni, Abdulrhman S Alshehri

**Affiliations:** 1 Clinical Practice, Faculty of Pharmacy, Northern Border University, Rafha, SAU; 2 Nursing, Al Baha University, Al Baha, SAU; 3 Critical Care, Dr. Soliman Fakeeh Hospital, Riyadh, SAU; 4 Emergency Medicine, Dr. Soliman Fakeeh Hospital, Riyadh, SAU; 5 Clinical Laboratory Science, College of Applied Medical Sciences, University of Hafr Al-Batin (UHB), Hafar Al Batin, SAU; 6 Biochemistry, King Abdulaziz University, Jeddah, SAU; 7 Medicine, Batterjee Medical College, Aseer, SAU; 8 Healthcare Management, College of Applied Medical Sciences, Shaqra University, Shaqra, SAU; 9 Clinical Pharmacy, Princess Nourah Bint Abdulrahman University, Riyadh, SAU; 10 Family Medicine, Armed Forces Hospitals, Taif, SAU; 11 Emergency, Al Hada Military Hospital, Taif, SAU

**Keywords:** antimicrobial resistance, bloodstream infection, clinical outcomes, gram-negative bacteremia, meta-analysis, mortality, saudi arabia

## Abstract

This study aimed to review the evidence systematically and quantify the association between antimicrobial resistance (AMR) in gram-negative bloodstream infections (GNBSI) and all-cause mortality in hospitals in Saudi Arabia. Following Preferred Reporting Items for Systematic Reviews and Meta-Analyses (PRISMA) guidelines and a PROSPERO protocol (CRD420251167703), databases (December 2010-October 2025) for observational studies comparing mortality in resistant versus susceptible GNBSI were searched. Risk of bias assessment using Risk of Bias in Non-randomized Studies of Interventions (ROBINS-I) was performed. Data pooling was achieved using a random-effects meta-analysis (Mantel-Haenszel method) incorporating the Hartung-Knapp adjustment to calculate pooled odds ratios (ORs) and 95% confidence intervals (CIs). Of 1,297 initial records, three retrospective cohort studies representing 19,056 patients satisfied the inclusion criteria. Risk of bias ranged from low to serious. Patients infected with resistant gram-negative pathogens demonstrated significantly higher odds of mortality compared to those with susceptible infections (pooled OR: 2.35; 95% CI: 2.30-2.41; p < 0.0001). Statistical heterogeneity was negligible (I² = 0.0%), but the small number of studies limits this finding. In this first systematic review and meta-analysis from Saudi Arabia, AMR in GNBSI was associated with a greater than twofold increase in the mortality odds. Although with limited and heterogeneous evidence, findings underscore the clinical burden, highlighting the need for antimicrobial stewardship, enhanced infection control measures, and large-scale prospective research to inform public health policies.

## Introduction and background

Bloodstream infections caused by gram-negative bacteria (GNBSI) are a critical and escalating threat to public health, contributing to morbidity, mortality rates, and medical costs [1,2]. Even in advanced healthcare environments, these infections are a leading cause of sepsis, carrying case fatality rates between 12% and 15% [3,4]. The clinical difficulties associated with GNBSI are compounded by the rising crisis of antimicrobial resistance (AMR) as resistance compromises the effectiveness of empirical and definitive therapies, resulting in treatment delays, prolonged hospital stays, and poorer patient outcomes [5,6].

Saudi Arabia is characterized by factors that accelerate the emergence and transmission of resistant pathogens, including high rates of antibiotic consumption, a dynamic healthcare system, and unique population movements associated with international events such as the Hajj and Umrah pilgrimages [7,8]. Surveillance studies in Saudi Arabia have documented an alarming prevalence of multidrug-resistant (MDR) gram-negative bacteria, as carbapenem resistance among Enterobacteriaceae has emerged as a threat, and high rates of resistance have been reported in non-fermenting pathogens, such as *Acinetobacter baumannii* and *Pseudomonas aeruginosa* [9-11]. Furthermore, high-risk clones, such as hypervirulent and MDR *Klebsiella pneumoniae* ST2096, have been identified as drivers of hospital outbreaks associated with severe clinical outcomes, including sepsis and elevated mortality [12].

Although numerous regional studies have characterized the epidemiological trends and molecular mechanisms of AMR, a synthesis correlating these resistance patterns with comparative clinical outcomes is absent. Most literature consists of descriptive prevalence studies or single-center analyses, which, although valuable, provide a fragmented understanding of the true clinical burden of resistance [13-15]. Understanding the precise quantitative impact of AMR on patient-centered outcomes, most critically mortality, is essential for informing empirical therapy guidelines, prioritizing antimicrobial stewardship interventions, and justifying investments in infection prevention and control infrastructure. A quantitative synthesis of the evidence is required to delineate the mortality risk attributable to resistance in this high-burden region of the world.

Therefore, given the current scarcity of comparative data, an exploratory systematic review and meta-analysis of observational studies from Saudi Arabia were conducted to quantify the association between antimicrobial resistance in GNBSI and all-cause mortality. The primary objective was to estimate the pooled odds of mortality among hospitalized patients (inpatients) with resistant GNBSI compared to those with infections caused by susceptible strains within Saudi Arabian tertiary and general hospital systems, excluding outpatient settings.

## Review

Methods

Protocol and Registration

This systematic review and meta-analysis was conducted and reported in accordance with the Preferred Reporting Items for Systematic Reviews and Meta-Analyses (PRISMA) 2020 statement [16]. The review protocol was registered with the International Prospective Register of Systematic Reviews (PROSPERO; CRD420251167703). Although a network meta-analysis was planned, the limited number and heterogeneity of the identified eligible studies precluded the formation of a connected evidence network. The analysis was restricted to a pairwise meta-analysis comparing resistant and susceptible infections.

Eligibility Criteria

Studies were selected for inclusion based on adherence to the Population, Exposure, Comparator, Outcome, and Study Design (PECOS) criteria. The target population consisted of hospitalized individuals of any age within Saudi Arabia diagnosed with a monomicrobial GNBSI confirmed by microbiology.

The exposure was defined as infection with a GNB isolate displaying a specific resistance profile (e.g., carbapenem-resistant, extended-spectrum β-lactamase (ESBL)-producing). These were measured against a control group infected by the same bacterial species but confirmed as susceptible to the relevant antibiotics by standard interpretive guidelines (e.g., European Committee for Antimicrobial Susceptibility Testing (EUCAST) and Clinical and Laboratory Standards Institute (CLSI)). The primary outcome was all-cause mortality, categorized as either 30-day or in-hospital death.

Eligible study designs included analytical observational studies (both prospective and retrospective cohort studies) that reported comparative outcome data. Studies focusing on polymicrobial BSI and non-bacteremic infections, or those that did not provide a clear, microbiologically confirmed definition of the resistance phenotype were excluded. Descriptive studies, case series without comparator groups, and narrative reviews were also excluded.

Information Sources and Search Strategy

A search of several electronic databases, specifically Scopus, Embase, the Cochrane Central Register of Controlled Trials (CENTRAL), and MEDLINE (accessed via PubMed), was conducted. The search was limited to English-language manuscripts published from December 2010 through October 2025. To ensure the literature coverage was exhaustive, supplementary search techniques, including manual scanning of regional medical journals and conference abstracts, as well as bidirectional citation screening (checking both reference lists and citing articles) of included studies and relevant reviews, were utilized. The search string integrated controlled vocabulary (Medical Subject Headings (MeSH) and Emtree) with a wide range of free-text terms related to gram-negative bacteremia, resistance patterns, and the Saudi Arabian healthcare setting. Detailed search strategies for each database, including all Boolean operators, are documented to ensure full replicability.

Study Selection

**Figure 1:**
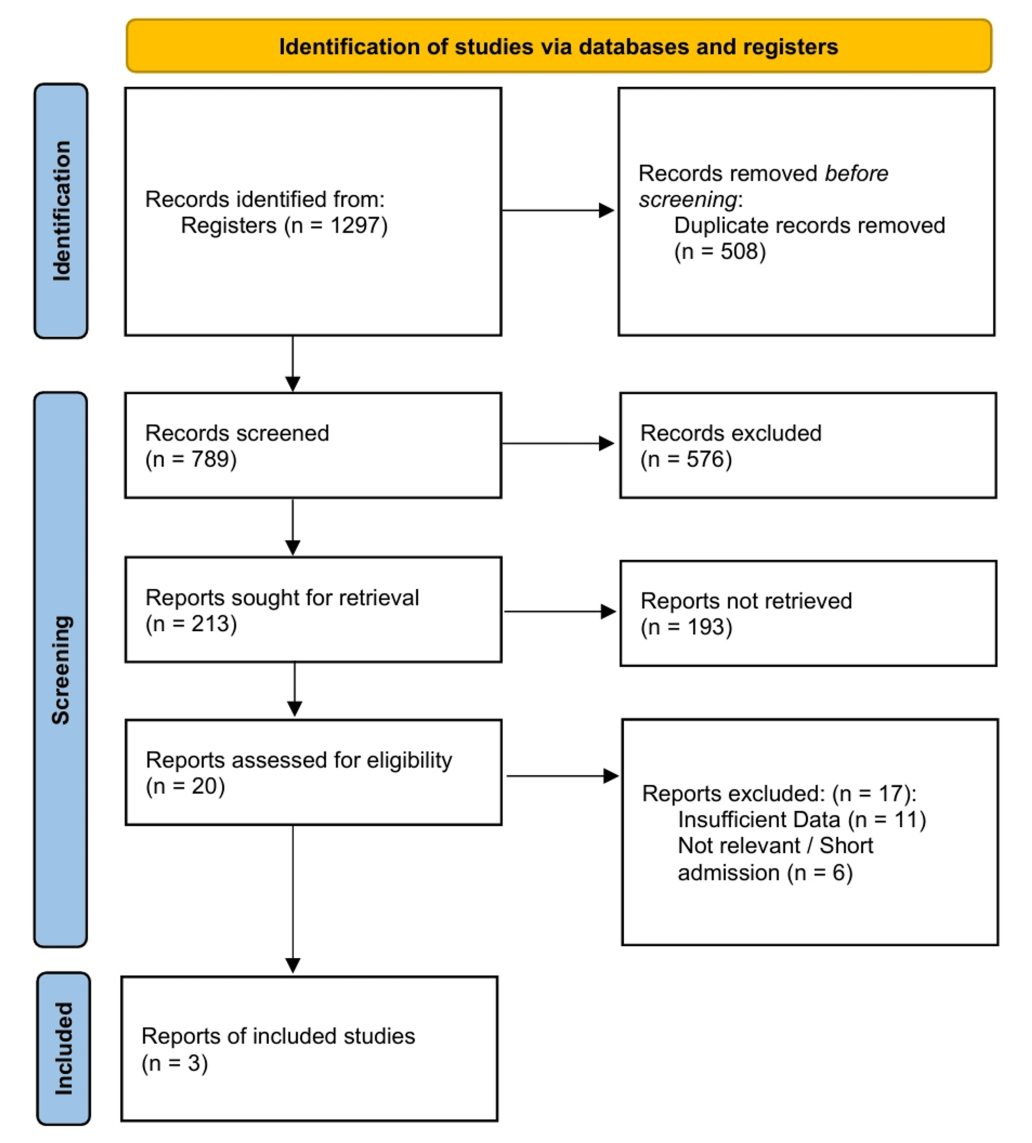
PRISMA Flow Diagram PRISMA: Preferred Reporting Items for Systematic Reviews and Meta-Analyses

The results of the literature search were deduplicated, while all titles and abstracts against predefined eligibility criteria were screened. The full texts of potentially relevant articles were retrieved and assessed in duplicate. The results of the study selection process are summarized in the PRISMA 2020 flow diagram (Figure 1).

Data Extraction

A standardized, pilot-tested data extraction form was used to collect relevant information from the included study, and data was extracted. Data was organized into four distinct categories: (i) study characteristics, comprising the first author, year of publication, study design, and clinical setting; (ii) patient population demographics, specifically sample size, mean age, and key comorbidities; (iii) microbiological definitions, detailing the criteria for the resistant (exposure) and susceptible (comparator) groups; and (iv) clinical outcome data, specifically the raw counts of all-cause mortality events and the total number of patients in each arm to facilitate odds ratio calculation.

Risk of Bias Assessment

Two independent reviewers from the research team independently evaluated the quality and potential bias of the selected observational studies. This assessment utilized the Risk of Bias in Non-randomized Studies of Interventions (ROBINS-I) instrument [17], which examines seven specific domains, including confounding, participant selection, intervention classification, deviations from intended interventions, missing data, outcome measurement, and reporting selection. Each domain was rated as having low, moderate, serious, or critical bias risk, culminating in an overall bias judgment for every study. Any discrepancies between reviewers were settled through consensus discussions.

Data Synthesis

Statistical computations were performed using R software (version 4.5.1, R Foundation for Statistical Computing, Vienna, Austria) utilizing the "meta" package (v8.2-1) [18].

The primary outcome of all-cause mortality was a dichotomous variable; therefore, the odds ratio (OR) was chosen as the summary effect measure. To harmonize the mixed resistance phenotypes identified across the included studies, disparate definitions (phenotypic carbapenem-resistant Enterobacteriaceae (CRE), genomic clones, and MDR profiles) were collapsed into a binary "Resistant" exposure variable for the quantitative synthesis. The number of events (deaths) and total patients from the resistant (exposed) and susceptible (comparator) groups of each study were extracted to construct 2 × 2 contingency tables.

Individual study ORs and pooled summary estimates were calculated using a random-effects model, which was selected a priori, as specified in our PROSPERO protocol, to account for the anticipated clinical and methodological heterogeneity across studies, which were expected to vary in terms of patient populations, specific pathogens, and healthcare settings [19]. The Mantel-Haenszel method was used to pool the data. To provide a more conservative and robust estimate of uncertainty, particularly given the small number of included studies, the 95% confidence interval (CI) for the pooled OR was calculated using the Hartung-Knapp-Sidik-Jonkman (HKSJ) method [20].

Statistical heterogeneity among the studies was evaluated using Cochran's Q test, with a p-value < 0.10 indicating significant variation. The extent of heterogeneity was gauged via the I² statistic, which estimates the proportion of variance across studies due to true differences rather than chance [21]. Because of the limited number of eligible studies, results from both the Q test and I² statistic were viewed with caution. The prediction interval, which estimates the expected range of true effects in similar future studies, was calculated to provide a representation of heterogeneity [22].

The results of the meta-analysis are presented in a forest plot, displaying the individual study ORs with their 95% CIs, the pooled OR with its 95% CI, and the corresponding prediction interval. The potential for small-study effects, including publication bias, was assessed by inspecting the asymmetry of the funnel plot and statistically using Egger's linear regression test for funnel plot asymmetry [23]. These methods are unreliable when fewer than 10 studies are included in a meta-analysis; thus, the results of this assessment were considered exploratory and interpreted with extreme caution [24].

Results

Study Selection

The initial database search yielded 1,297 records. Following the elimination of 508 duplicate entries, the titles and abstracts of the remaining 789 unique records were screened. During this preliminary phase, 576 records were discarded as they did not satisfy the inclusion criteria. The full text of 213 articles were retrieved and reviewed. From these, 193 reports were unavailable, leaving 20 full-text articles for detailed eligibility assessment, of which 17 were excluded. The primary reasons for exclusion at this stage were the provision of insufficient data to extract a 2 × 2 contingency table for the outcome of interest (n = 11) and a focus on an irrelevant population or context (n = 6).

Three unique studies met the inclusion criteria and were included in the systematic review and meta-analysis. The complete study selection process, including the number of records at each stage, is detailed in the PRISMA flow diagram (Figure 1) [16].

Study Characteristics

Three retrospective cohort studies encompassing 19,056 patients met the full inclusion criteria and were included in the quantitative synthesis [7,12,13]. The key characteristics of these studies are presented in** **Table 1.

**Table 1:** Characteristics of Included Studies AMR: antimicrobial resistance, BSI: bloodstream infection, MDR: multidrug-resistant, RoB: risk of bias

Study ID	Alshaddadi et al. [7]	Hala et al. [12]	Alsaadi et al. [13]
Publication year	2025	2024	2024
Study period	2015-2024	2014-2022	2015-2022
Study design	Retrospective cohort	Retrospective cohort	Retrospective cohort
Setting	Multicenter (24 hospitals)	Single center (tertiary)	Multicenter (5 hospitals)
Population	Patients with various AMR infections	Patients with MDR K. pneumoniae BSI	Patients with M. morganii BSI
Resistant group (exposure)	Carbapenem-resistant Enterobacteriaceae (CRE)	K. pneumoniae ST2096 clone	MDR M. morganii
Susceptible group (comparator)	Carbapenem-susceptible Enterobacteriaceae	Non-ST2096 clones	Susceptible M. morganii
Outcome	30-day mortality	In-hospital mortality	In-hospital mortality
Sample size (number)	18,742	239	75
Overall RoB	Low (+)	Moderate (!)	Serious (-)

The included studies were published between 2024 and 2025, with patient data collection periods spanning 2014-2024, and the scope and scale of these studies varied. Alshaddadi et al. conducted a large, national-level, multicenter cohort study across 24 hospitals, providing data on 18,742 patients with various antimicrobial-resistant infections [7]. In contrast, Hala et al. conducted a focused, single-center genomic epidemiology study on 239 patients with MDR *Klebsiella pneumoniae* [12], and Alsaadi et al. performed a smaller multicenter analysis of 75 patients with *Morganella morganii* bacteremia across five hospitals [13].

Clinical heterogeneity was evident across the included populations and specific resistance phenotypes, as Alshaddadi et al. provided data comparing the outcomes of patients with carbapenem-resistant Enterobacteriaceae (CRE) [7]. Hala et al. compared the mortality between patients infected with the hypervirulent MDR *K. pneumoniae* ST2096 clone and those infected with non-ST2096 clones [12]. Alsaadi et al. provided outcome data on patients with *M. morganii* bacteremia stratified by resistance profile [13]. All three studies reported all-cause mortality (either in-hospital or at 30 days) as a primary or secondary outcome, which enabled quantitative synthesis.

The overall risk of bias for the included studies, assessed using the ROBINS-I tool, was judged as low for one study [7], moderate for one study [12], and serious for one study [13], with key concerns related to potential confounding and the handling of missing data in the latter two.

Risk of Bias

**Figure 2:**
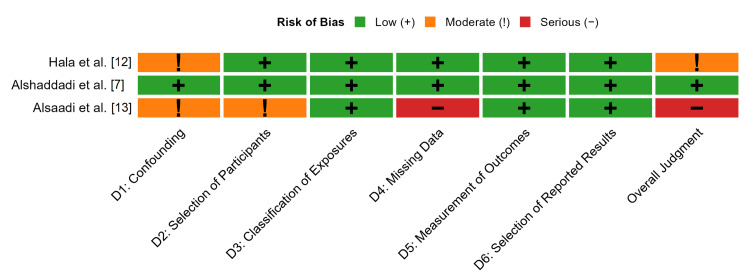
Risk of Bias Assessment for Individual Studies

**Figure 3:**
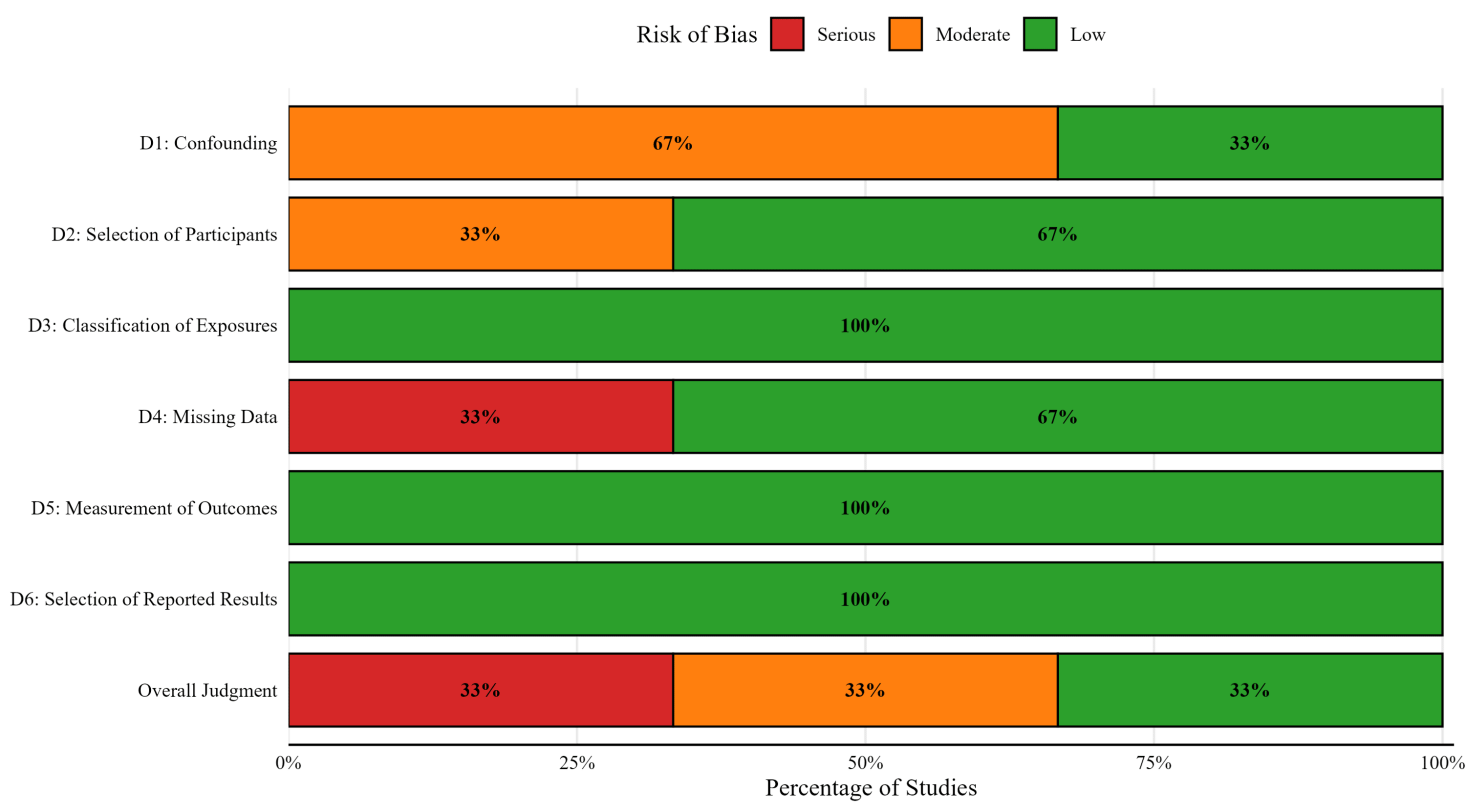
Summary of Risk of Bias Assessment Across All Studies

The methodological quality and risk of bias of the three included observational studies were systematically evaluated using the ROBINS-I tool [17]. A detailed summary of the domain-by-domain judgments and overall risk of bias for each study is presented in Figure 2, while a summary of these judgments across all studies is shown in Figure 3.

The evidence had a variable risk of bias. Alshaddadi et al. was judged to be at a low risk of bias, demonstrating robust methodological approaches, including the use of multivariable survival analysis to control for key confounders and appropriate statistical methods (multiple imputation) to handle missing data [7].

Hala et al. was judged to have a moderate risk of bias [12]. Although the study used advanced methods for exposure classification (whole-genome sequencing), the risk of bias due to confounding was moderate because the covariates adjusted for in the clinical outcome analysis were not detailed, raising the possibility of residual confounding.

Alsaadi et al. was judged to be at a "serious" risk of bias [13] because of the significant concerns in multiple domains, as the small sample size (n = 75) led to a moderate risk of bias in participant selection and limited the reliability of statistical adjustments for confounding. A serious risk of bias was assigned to the domain of missing data, as the study provided no information on how missing values were managed, which can introduce substantial bias in a small dataset.

Across all included studies, the risk of bias was low in the domains of classification of exposures (as resistance was microbiologically confirmed), measurement of outcomes (mortality was an objective endpoint), and selection of reported results.

Synthesis of Results

The three eligible studies, comprising 19,056 patients, provided sufficient data for inclusion in the quantitative meta-analysis of all-cause mortality [7,12,13].

**Figure 4:**
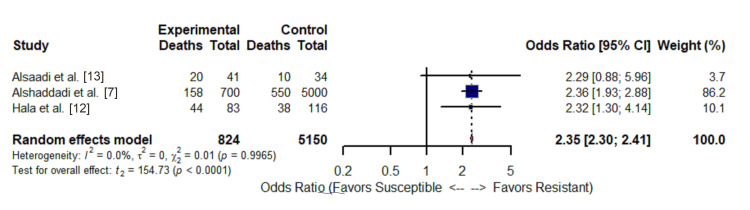
Publication Quality Forest Plot The pooled estimate was influenced by the large Alshaddadi et al. study, which contributed 86.2% of the weight in the random-effects model.

As shown in the forest plot (Figure 4), the random-effects meta-analysis demonstrated that infection with a resistant gram-negative bacterium was associated with a statistically significant and clinically substantial increase in the odds of mortality compared with infection with a susceptible strain (pooled odds ratio (OR): 2.35; 95% CI: 2.30-2.41; p < 0.0001). The point estimates from all three individual studies indicated a direction of harm for the resistant group, but the association was statistically significant in two cohorts. The pooled estimate was influenced by the large Alshaddadi et al. study, which contributed 86.2% of the weight in the random-effects model.

An assessment of statistical heterogeneity indicated no significant variability between studies (I² = 0.0%; Cochran's Q p = 0.99). However, it is critical to note that the I² statistic and the Q test have very low statistical power with only three studies. Therefore, the observed I^2^ of 0.0% is not evidence of true homogeneity [21], and the high precision of the pooled confidence interval (95% CI: 2.30-2.41) should be interpreted with caution. This precision is largely driven by the overwhelming statistical weight (86.2%) of the Alshaddadi et al. [7] dataset, meaning the pooled estimate effectively restates the findings of this single large cohort rather than representing a balanced average across diverse settings. Clinical and methodological heterogeneity across the studies was considerable, as reflected in the different pathogens, study sizes, and settings used.

**Figure 5:**
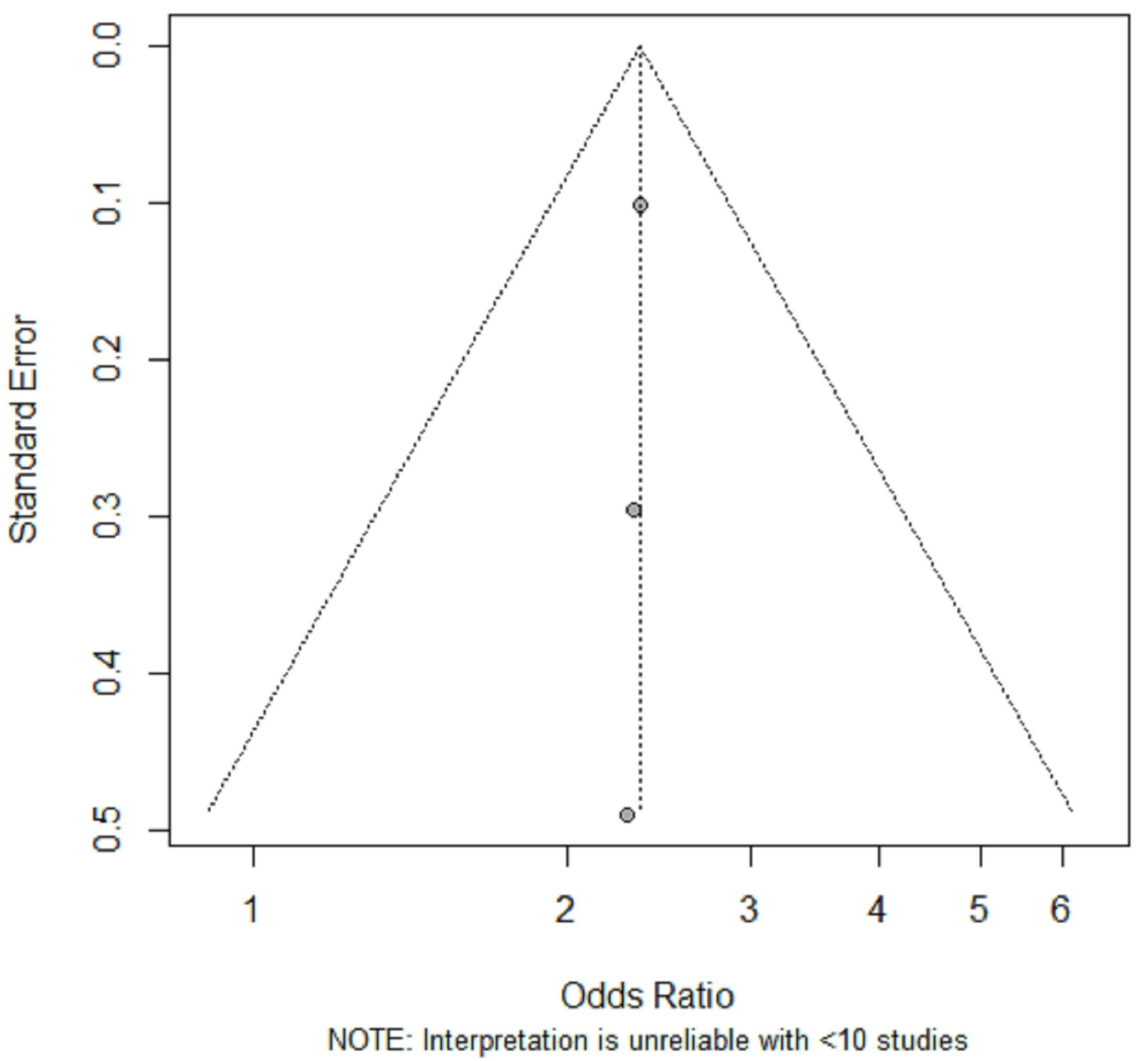
Publication Bias Funnel Plot

The potential for small-study effects, including publication bias, was also evaluated. The visual inspection of the funnel plot was limited by the small number of studies included (Figure 5), while a formal statistical test for asymmetry was not performed because such tests are considered unreliable and potentially misleading with fewer than 10 studies [24]; therefore, the presence of reporting bias could not be meaningfully assessed or excluded.

Reporting Bias

The potential for reporting bias and other small-study effects was evaluated using a funnel plot (Figure 5) and Egger's linear regression test. With only three studies included in the meta-analysis, the plot was too sparsely populated to allow for a meaningful visual assessment of asymmetry. Consistent with visual inspection, the R console reported a warning that the number of studies was insufficient for formal testing of small-study effects.

It must be emphasized that with fewer than 10 studies, both funnel plots and their associated statistical tests have very low power and are not recommended for detecting publication bias [24]; therefore, while no formal evidence of reporting bias was detected in this analysis, its presence cannot be reliably ruled out because of the limited number of studies available for this synthesis.

Discussion

This study is the inaugural systematic review and meta-analysis providing a quantitative synthesis of how antimicrobial resistance impacts GNBSI-related mortality within Saudi Arabia. The analysis of three cohort studies involving more than 19,000 patients found that infection with resistant gram-negative bacteria is linked to a greater than twofold rise in the odds of all-cause mortality relative to susceptible infections (pooled OR: 2.35; 95% CI: 2.30-2.41). This finding was statistically significant and directionally consistent across all included studies, highlighting the substantial and clinically significant burden of resistance on patient outcomes in the region.

The magnitude of this association aligns with findings from the literature, which have identified antimicrobial resistance as an independent predictor of adverse outcomes in GNBSI [4,13]. The primary mechanism underlying this increased mortality is the higher probability of initial inappropriate or delayed effective antimicrobial therapy, which has been identified as a key modifiable risk factor for death in patients with severe infections [5,7]. The pathogens included in this synthesis, ranging from specific high-risk clones such as *K. pneumoniae* ST2096 to broader categories such as carbapenem-resistant Enterobacteriaceae, underscore that this detrimental effect is not confined to a single organism but is a generalized consequence of the resistance phenotype, which limits therapeutic options and complicates clinical management [12,13].

Strengths

The strengths of this review include its adherence to a pre-registered PROSPERO protocol, comprehensive search strategy, and rigorous risk of bias assessment using the ROBINS-I tool. A statistically conservative estimate was provided by applying a random-effects model with the Hartung-Knapp adjustment that accounted for the small number of studies. This study addresses a critical evidence gap by providing the first pooled quantitative estimate of the mortality impact of AMR in GNBSI in the Saudi Arabian context, a known hotspot for AMR [8,11].

Conceptual Heterogeneity of Resistance Definitions

It is acknowledged that this meta-analysis pools fundamentally non-equivalent exposures, ranging from phenotypic classifications (CRE) to specific genomic high-risk clones (MDR *K. pneumoniae* ST2096). While this violates the assumption of strict exchangeability required for a precise causal effect size, this pooling was necessary to generate an exploratory baseline for the region. The pooled OR should be interpreted not as a homogeneous biological effect, but as a directional signal of harm. This approach admits residual confounding, specifically the interplay between resistance and virulence, but confirms that regardless of the specific mechanism, the presence of resistance markers consistently correlates with doubled mortality odds in this setting.

Limitations

However, the most significant limitation is the small number of studies included (N = 3), as this scarcity of eligible studies constrains the precision of the pooled estimates and the generalizability of our findings. Despite low statistical heterogeneity (I² = 0.0%), there was considerable clinical and methodological heterogeneity across studies in terms of specific pathogens, patient populations, and study settings. The low I^2^ value is a statistical artifact resulting from the low power of the test with few studies rather than true homogeneity [6]. The pooled estimates are interpreted as an exploratory signal of harm rather than a definitive effect size, a narrative approach consistent with Cochrane guidance on the synthesis of sparse and heterogeneous data [19].

The risk of bias assessment revealed variable quality, with one study judged to be at serious risk of bias due to concerns about confounding and missing data, which weakened the overall certainty of the evidence base. Finally, a planned network meta-analysis could not be performed due to insufficient data, preventing a comparative analysis of different resistance phenotypes.

Implications

The findings have critical implications for clinical practice and public health policy in Saudi Arabia. The observed signal of harm associated with resistance validates the urgent need to strengthen antimicrobial stewardship programs to preserve existing agents. Also, this evidence reinforces the importance of robust infection prevention measures to halt the transmission of MDR organisms. Investment in rapid diagnostic technologies is paramount to minimize the time to effective therapy, thereby mitigating the mortality burden.

From a research perspective, this review highlights profound data deficits as there is a need for large-scale, multicenter, prospective cohort studies in Saudi Arabia that link detailed microbiological data to robust clinical outcomes as these studies would provide a precise and generalizable estimate of the mortality burden and enable the investigation of specific resistance mechanisms and patient subgroups that would generate high-quality evidence required to inform national treatment guidelines, guide public health interventions, and populate a future network meta-analysis to compare the relative impact of different resistance phenotypes.

## Conclusions

In this first systematic review and meta-analysis to quantitatively assess the impact of antimicrobial resistance on GNBSI outcomes across healthcare facilities in Saudi Arabia, it is found that infection with a resistant pathogen was associated with a greater than twofold increase in the odds of mortality. However, this finding is drawn from limited and heterogeneous evidence; therefore, the estimate should be interpreted with caution. This evidence gap underscores the imperative for high-quality, large-scale national surveillance and prospective cohort studies to precisely quantify the clinical burden of AMR and inform evidence-based antimicrobial stewardship and infection control policies in the Kingdom.
